# Oral Candidiasis in a Diabetic Patient Wearing Bar-Retained Provisional Overdenture: Clinical Case Report

**DOI:** 10.3390/reports7040096

**Published:** 2024-11-11

**Authors:** Christopher Diego Nicholson-Becerra, Mónica Orozco-Gallardo, Arturo Cisneros-Moya, Evangelina Gutiérrez-Cortés, Andréa Dolores Correia Miranda Valdivia

**Affiliations:** 1 Faculty of Dentistry, Autonomous University of Guadalajara, Guadalajara 44160, Jalisco, Mexico; christopher.nicholson@edu.uag.mx (C.D.N.-B.); monica.orozco@edu.uag.mx (M.O.-G.); arturo.moya@edu.uag.mx (A.C.-M.); 2 Department of Oral Pathology, Faculty of Dentistry, Autonomous University of Guadalajara, Guadalajara 44160, Jalisco, Mexico; evangelina.gutierrez@edu.uag.mx; 3 Department of Specialized Dentistry, Faculty of Dentistry, Autonomous University of Guadalajara, Calle Escorza 526, Colonia Americana, Guadalajara 44160, Jalisco, Mexico

**Keywords:** oral candidiasis, denture-related stomatitis, diabetes, bar-retained implants

## Abstract

**Background and Clinical Significance**: Denture stomatitis is a clinical manifestation of oral candidiasis, often seen in individuals wearing removable dentures that lead to the formation of sub-prosthetic stomatitis. This is particularly common in maladjusted appliances that have been in use for many years. Studies have shown that patients with systemic diseases such as diabetes, or other medical complexities, have a higher likelihood of developing denture stomatitis. To address this problem, the use of implant-retained dentures with different types of attachments has been introduced, providing increased comfort and hygiene for edentulous patients. However, this solution is not without its own set of challenges, being that the prolonged contact with mucosal surfaces can lead to challenges in cleaning and managing plaque depending on the attachment. **Case Presentation:** In this clinical case report, we present a female patient who developed bar-retained prosthetic stomatitis induced by oral candidiasis a few months after receiving her provisional prosthesis. **Conclusions:** In conclusion, this case report emphasizes the need to consider both systemic and local factors when preventing and treating denture stomatitis. By understanding the risk factors involved, healthcare professionals can provide their patients with the best possible care, helping to reduce the prevalence of oral candidiasis in individuals who use implant-retained dentures.

## 1. Introduction and Clinical Significance

Implant-supported overdentures are praised for their stability, retention, and comfort. Nonetheless, they are susceptible to the same infectious complications as conventional overdentures and implants, such as denture-related stomatitis and peri-implantitis. Specifically, bar-retained overdentures may create difficulties in cleaning and controlling plaque due to prolonged contact with mucosal tissues. Denture stomatitis (DS), a prevalent condition among denture wearers, is marked by inflammation and redness in the mucosal areas in contact with the denture, affecting between 20% and 67% of users [1,2].

DS is a multifactorial condition, and like any disease, effective treatment relies on understanding the pathogenic microbial species involved. Candida albicans (CA) was first recognized as a potential causative agent of DS in 1936 and is now considered the most frequently implicated species [3]. Despite this, the etiology of denture stomatitis is not fully understood. It is also reported in studies that there is a higher incidence of denture stomatitis and proliferation of oral candidiasis (OC) in patients with systemic diseases, which in this case is diabetes mellitus (DM) [4,5].

DM patients often take systemic medications like antihypertensives and diuretics, which can reduce saliva production and encourage CA biofilm buildup [6]. Poor oral hygiene, combined with the difficulties of cleaning and maintaining implant-retained overdentures depending on the type of attachment (bar retained, locator, ball retained, etc.), allows biofilm to thrive and adhere to acrylic surfaces, leading to denture stomatitis [7]. Overdentures on bars offer many advantages for patients with a severely resorbed edentulous ridge, including having low maintenance costs [8]. However, there is some evidence indicating that solitary attachments are less expensive than bar attachments on the manufacturing side [9]. In addition, there is some evidence that states that solitary attachments, in this case, the locator system, are easier for the patient to clean compared to a bar attachment, resulting in soft tissue and the bone being healthier [8,9]. DS can be clinically identified through the ex-amination of inflamed and red mucosa beneath maxillary complete dentures. Key signs include white patches, mucosal thickening, and the presence of fungal growth on the denture itself [10].

The classification system proposed by Newton in 1962 is the most utilized, distinguishing three types of denture stomatitis: (1) pinpoint hyperemic spots, (2) diffuse redness of the denture-supporting tissues, and (3) papillary hyperplasia [9]. In contrast, Budtz-Jorgensen and Bertram in 1970 used slightly different terms for similar conditions: (1) simple localized inflammation, (2) simple diffuse (generalized) inflammation, and (3) granular inflammation [11].

DS induced mainly by the proliferation of CA is a chronic condition that challenges the prosthetic phase of treatment, and the cleanability of these implant-retained overdentures, with its attachments, is critical for its maintenance and longevity. Treatment primarily focuses on administering local and systemic antifungal medications, along with reducing or eliminating denture-associated biofilm including using well-adjusted overdentures along with a different type of implant attachment to improve hygiene maintenance [12]. Nevertheless, improving oral hygiene, treating systemic conditions, and having frequent check-ups with one’s dental provider in addition to providing antifungal treatment have been documented to be effective in treating oral candidiasis [13,14]. In this clinical case report, we present a female patient who developed bar-retained prosthetic stomatitis induced by oral candidiasis a few months after receiving her provisional prosthesis.

## 2. Case Presentation

**Figure 1:**
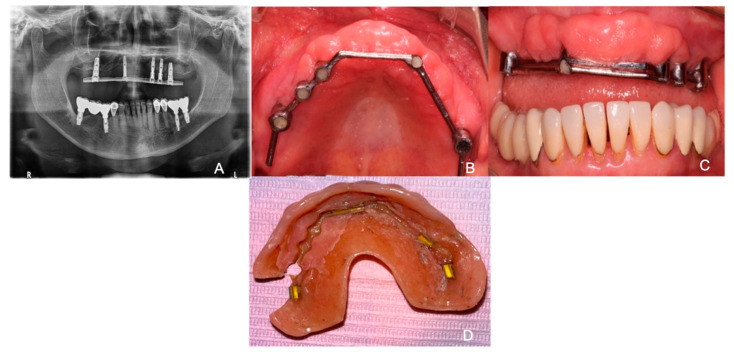
(**A**) Panoramic radiographic image displaying the failed bar-retained attachment. (**B**) Bottom of the palate with white appearance and generalized inflammation. (**C**) Anterior part of maxilla with multinodular oval-shaped tissue. (**D**) Interior part of the overdenture with presence of plaque and a fracture in the posterior left side.

A 66-year-old female patient attended the diagnostic department with the primary concern being her fractured prosthesis and discomfort on the area where her provisional over-denture was placed. The patient reports being hypertensive and controlled type II diabetes. Additionally, it was noted that the patient had stomach ulcers and was a frequent smoker. The informed consent was obtained, and we filled in the medical history of the patient. A panoramic X-ray was performed for a first evaluation of the situation of the implants and the bar (Figure 1A). On intraoral examination, it was observed that there was the presence of white plaque on the bottom of the palate with a diameter of 5–6 mm, with defined edges and no increase in volume in which it would come off by scraping with gauze (Figure 1B). In addition, we observed a multinodular and oval-shaped lesion of 4–6 mm in diameter with well-defined borders in the anterior region of the maxilla on an erythematous background and semi-pedunculated base (Figure 1C). In the extra-oral examination, it was observed on the prosthesis that the bar-retained overdenture was fractured on the left posterior side along with whitish areas in the interior part of the overdenture that may have indicated plaque and fungal colonization (Figure 1D). Therefore, the patient was referred to the Oral Medicine, Periodontics, and Prosthodontics Department.

**Figure 2:**
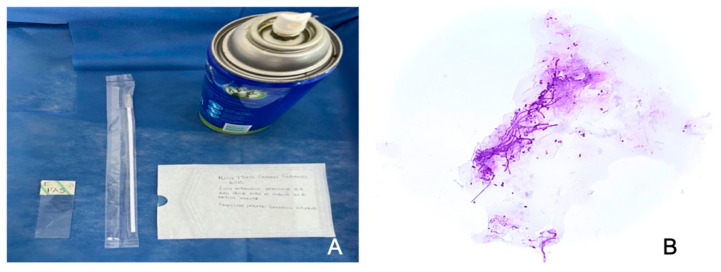
(**A**) Equipment used on the exfoliative cytology. (**B**) Microscopic image: glass slide stained with PAS observed under a microscope and we observe detached epithelial cells that contain elongated pseudo hyphae that indicates a colonization of the candida fungus.

The patient underwent exfoliative cytology during her diagnostic examination, testing positive for CA (Figure 2). She was treated with 20 milligrams of Miconazole oral gel (Alpharma, Mexico City, Mexico), applied twice a day. The results have been reviewed in the Department of Oral Medicine, and her progress is very good, particularly for subplaque candidiasis and hydration of the mouth. Supportive treatment with Nystatin suspension (Alpharma, Mexico City, Mexico) has been prescribed for an additional 15 days.

**Figure 3:**
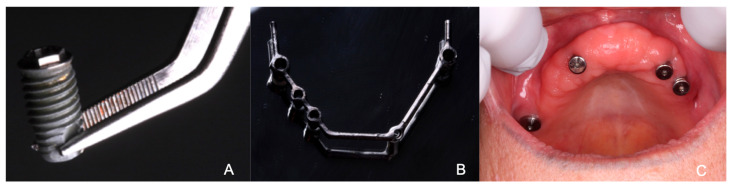
(**A**) Implant from site 26 that was dislodged. (**B**) Hader bar attachment after being removed. (**C**) Maxilla with healing abutments placed.

After two weeks, the patient presented to the Prosthodontic department for the removal of the upper implant-supported bar. The resin plugs were removed, and the bar was unscrewed. Upon removing the bar from the implant at site 26, purulent discharge was noted at the gingival margin. Healing abutments were placed (Nobel WP (Zurich, Switzerland) at site 16 and Biohorizons (Birmingham, AL, USA) 4 mm at sites 13, 23, and 24). The implant at site 26 was only fibrointegrated, and upon attempting to place a healing abutment, the implant dislodged from the alveolus. The periodontist resident irrigated the area thoroughly. The patient’s previous prosthesis was repaired and a soft reline was placed over the healing abutments. An alginate impression was taken to start the process of an upper transitional prosthesis (Figure 3).

**Figure 4:**
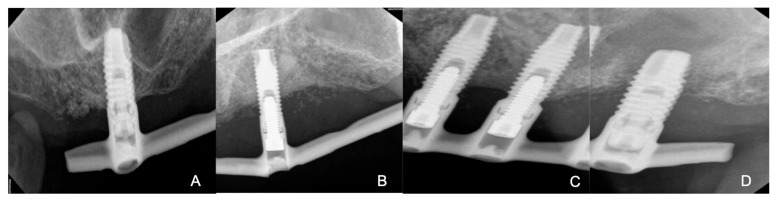
(**A**) Implant with good peri-implant health. (**B**) Implant with good peri-implant health. (**C**) Implant with peri-implantitis and exposed threads. (**D**) Implant with peri-implantitis and mobility.

After a week, upon intraoral examination conducted in the Periodontics Department, radiographic images previously taken before the removal of the Hader bar attachment were reviewed. It was found that three implants with a probing depth (PD) of 4 mm showed bleeding without radiographic bone loss or suppuration (Figure 4A,C). One implant with a PD of 7 mm showed bleeding and suppuration in addition to radiographic bone loss greater than 2 mm (Figure 4B) and one implant with a PD of 11 m showed bleeding, suppuration, as well as mobility and bone loss around the entire perimeter of the implant (Figure 4D). This implant was chosen for extraction (Figure 4D) due to poor prognosis. The treatment of choice for the implant (Figure 4C) was implantoplasty.

**Figure 5:**
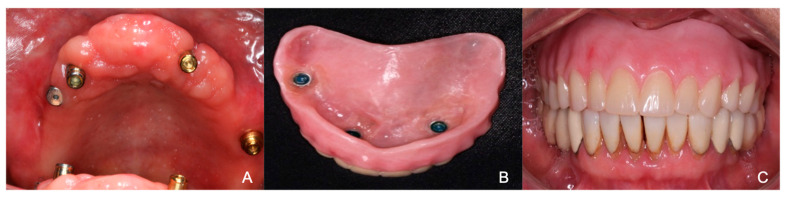
(**A**) Implants ready to receive the provisional overdenture without the metal bar and instead locator attachments were placed (one healing abutment on the right side of the maxilla since tissue was continuing healing). (**B**) Interior surface of the new heat-activated acrylic overdenture after try-in. (**C**) Frontal view of the overdenture in occlusion on patient’s mouth.

After one week, the patient presented to the Periodontics Department for the removal of threads from the upper right implant due to peri-implant bone loss and bleeding on probing, with a depth of 9 mm. The procedure began with a 0.12% chlorhexidine rinse, followed by the administration of 2% mepivacaine using two cartridges with infraorbital and nasopalatine techniques via a short needle. The gingival area was trimmed using an external bevel with a #15C blade, and a flap was raised. A diamond bur was then used to polish and remove the implant threads, followed by suturing with a 5-0 nylon. Some weeks after, the prosthodontic resident changed the type of attachments into a locator system to facilitate hygiene and maintenance for the patient. In addition, a new provisional overdenture was fabricated (Figure 5).

## 3. Discussion

Individuals who wear dentures are particularly susceptible to Candida-associated denture stomatitis (CADS) because the usual oral commensal Candida species can transform into a pathogenic form when conditions become favorable in immune-compromised patients [15]. Oral candidiasis is a clinical diagnosis, exfoliative cytology is a straightforward, safe, and dependable technique that we use for microscopically analyzing cells that have been naturally shed or sloughed off from the mucosal surface [16]. It consists of observing under a microscope the morphology of the superficial epithelial cells after their collection, fixation, and Periodic Acid-Schiff (PAS) staining in this case.

The PAS stain is frequently used to identify glycogen. This technique is based on periodic acid’s ability to oxidize carbohydrate molecules, which then react with the aldehyde groups to produce a detectable color change [17].

Further research is essential for distinguishing between diagnoses and addressing cases that are resistant to antifungal treatments. In a recent study by Martorano et al., diabetic patients are as likely as non-diabetic individuals to develop oral candidiasis. However, they are more prone to experiencing denture stomatitis than healthy individuals. This can be because of certain diabetic medications on the environment in the mucosa-denture interphase, especially the salivary secretion rate and components. In addition, wearing dentures can create an anaerobic microenvironment that promotes the growth of candida due to factors such as reduced salivary flow, low Ph, poor oral hygiene management, improperly fitted dentures, or enhanced adherence of candida to acrylic [18,19]. This anaerobic microenvironment is also present in overdentures due to the prolonged contact with the mucosa and the implant attachments.

Implant attachments, can be classified into two groups: bar attachment and solitary attachment systems. The choice of attachment is based on the experience of the practitioner and clinical outcomes. Solitary type attachments, including ball, magnet and Locator attachments have advantages such as simpler oral hygiene maintenance and narrow interarch space. On the other hand, a parallel implant placement is required, and the stability of the overdenture is lower than the bar type. Bar attachments—the example being the Hader bar in this case—the Dolder bar, and milled bar, can evenly distribute loading imposed at mastication, and the placement inclination is less limited [20].

In a 2008 study, the main complications in the bar-attachment group were hygiene complications. According to their patients in the bar-attachment group, it is very difficult to clean the periabutment zone. But after a year in function, they developed their cleaning skills and problems have disappeared [21]. In the solitary attachment, the locator system was introduced in 2001 by Zest Anchors Company in Escondido, CA, which does not use the splinting of implants. This type of attachment has become popular ue to its self-aligning property, simplicity of use, and minimal space requirement of male inserts within the denture [22].

Chaware et al., published a systematic review and meta-analysis comparing different attachments, the bar attachments reported moderate tissue reaction in the form of mucosal changes, gingival inflammation, and bone resorption. The locator attachments require higher maintenance and repair [23], mainly due to nylon’s high rate of deformation and damage, which is linked to high number of control appointments.

Kilic et al., evaluated the colonization of candida species along with denture related stomatitis in bar and locator-retained overdentures. All the patients wearing bar-retained overdenture developed DS, while patients using locator-retained overdentures it developed in 71.4%. In addition, candida species cfu values were significantly higher in bar-retained over-denture wearers compared to locator-retained overdenture wearers along with gingival and plaque indexes [24]. This may be due to the narrow space between the bar and the tissue, creating some difficulty in obtaining proper cleaning and maintenance of the area.

In this case, we treat these lesions with antifungical medication, improving oral hygiene and at the same time providing a new overdenture. The Diseases Society of America (IDSA) guidelines states that topical antifungal therapy should be the first line of therapy for mild oral candidiasis [25]. Although, topical agents like nystatin, amphotericin B, and clotrimazole have limitations, such as short retention on oral mucosa and the sugar content on the topical agents that can affect the oral cavity and patients suffering with systemic diseases, still preferred over systemic ones due to renal and hepatotoxicity associated with [11].

Miconazole can damage the integrity of the fungal cell membrane, alter fungal adherence, and inhibit the formation of germ tubes and mycelia. Miconazole has potent broad-spectrum activity against many candida species [26]. In our case, we reinforce our antifungal medication with oral suspension of nystatin, which is, a polyene antibiotic, interacts with the ergosterol in the fungal cell membrane making it porous and vulnerable to lysis, thus exerting its antifungal effect [15]. IDSA recommends the usage of nystatin suspension having a concentration of 100,000 IU/mL with a dosage of 4–6 mL four times a day for mild oral/oropharyngeal candidiasis [25]. Therefore, in this case, the use of miconazole gel or oral jelly and nystatin oral suspension turns out to be a viable treatment of denture stomatitis induced by candida because they are effective antifungal agents and do not produce significant adverse effects [26].

In a 2023, systematic review and meta-analysis determining the effective cleaning and disinfection of removable prostheses, the recommended approach includes brushing the prosthesis, followed by daily immersion in warm water (~37 °C) with cleaning tablet (Efferdent, Polident, Denture Brite, etc.) or soak the denture in 0.5% sodium hypochlorite (El Crisol, Mexico City, Mexico) for 10 min, then place it in a solution of alkaline peroxide tablets and water overnight. If you cannot clean the denture overnight, it is advisable to keep it dry to help prevent Candida colonization [27].

Similar to OC, the primitive causative factor for peri-implant diseases is dental plaque. They harvest especially, Gram-negative anaerobic bacteria compared with healthy sites [28]. According to the Classification of Periodontal and Peri-implant Diseases and Conditions 2018, the identification of Peri-implantitis can be established based on the following indicators: (1) detection of inflammation in the vicinity of peri-implant tissue, (2) observation of bone loss through radiographic evidence subsequent to the initial healing phase, and (3) discernible elevation in probing depth in comparison to the probing depth following the placement of the prosthetic reconstruction [29].

The management of peri-implant diseases can be categorized into three main approaches: (1) Non-surgical methods involving mechanical debridement, antiseptics, and antibiotics; (2) Surface decontamination techniques utilizing lasers and chemical agents such as citric acid, ethylenediaminetetraacetic acid (EDTA), hydrogen peroxide, and saline; and (3) Surgical interventions including air power abrasive procedures, resective surgery, and regenerative surgery [21]. In this patient’s case, applied implantoplasty, or mechanical modification of the implant surface, has been proposed as an alternative treatment for peri-implantitis. This procedure involves mechanically removing the implant threads to create a smooth surface that is less prone to plaque accumulation and subsequent reinfection. The surface is shaped using diamond burs as well as Arkansas and silicone polishers [30].

From a restorative perspective, the use of an implant-retained overdenture regardless of implant attachment has shown significant effectiveness in enhancing both oral health and nutrition in patients. When compared to a complete denture, an overdenture leads to a notable improvement of 20% in chewing efficiency [31]. Moreover, the maximum occlusal force in denture patients can increase by as much as 300% with the support of an implant-supported prosthesis [32].

## 4. Conclusions

This case report highlights the importance of comprehensive oral health management, including hygiene and oral health education, and fabrication of a new provisional overdenture with implant attachment to facilitate hygiene. Also, is important to emphasize that the follow up on any procedure is of great relevance because it will help not only to observe the satisfactory evolution of the lesion, but will also help to keep an eye out for any possible complications. If any complications occur, we will be able to provide an opportune treatment.

The prognosis for acute and most chronic candidiasis cases is favorable, with no clear link between chronic candidiasis and precancerous conditions. However, the invasion of the epithelium by Candida organisms and subsequent proliferation could potentially contribute to neoplastic transformation. Patient motivation to maintain proper hygiene is one of the most important indicators for treatment success. The proper use of diagnostic, comprehensive oral health management, follow-up treatments, and multidisciplinary teamwork are essential to successful treatments with implant-retained dentures.

## Data Availability

The original data presented in the study are included in the article, further inquiries can be directed to the corresponding author.
